# Chronic stress and cognitive dysfunction in myalgic encephalomyelitis/chronic fatigue syndrome: HPA axis dysregulation and hippocampal plasticity

**DOI:** 10.3389/fnins.2026.1814098

**Published:** 2026-04-22

**Authors:** Hailan Kang, Tianrui Shao, Yuqing Shi, Shilei Wang, Huazhong Xiong, Xuanyan Jin, Jixiang Ren

**Affiliations:** 1 College of Traditional Chinese Medicine, Changchun University of Chinese Medicine, Changchun, Jilin, China; 2 Northeast Asia Research Institute of Traditional Chinese Medicine, Changchun University of Chinese Medicine, Changchun, Jilin, China; 3 The Affiliated Hospital of Changchun University of Traditional Chinese Medicine, Changchun, Jilin, China; 4 Guang’anmen Hospital, China Academy of Chinese Medical Sciences, Beijing, China

**Keywords:** chronic stress, cognitive dysfunction, hippocampus, hypothalamic-pituitary-adrenal axis, myalgic encephalomyelitis/chronic fatigue syndrome

## Abstract

Cognitive dysfunction is a common and disabling clinical feature of myalgic encephalomyelitis/chronic fatigue syndrome (ME/CFS), often described by patients as “brain fog.” These symptoms typically manifest as difficulties in attention, memory, and concentration. Chronic stress has been proposed as an important contributing factor in ME/CFS. The hypothalamic-pituitary-adrenal (HPA) axis plays a central role in the stress response, and prolonged adverse stress may contribute to HPA axis dysregulation, including altered cortisol rhythmicity and impaired negative feedback regulation. Such dysregulation may be associated with cognitive dysfunction in ME/CFS through mechanisms involving neuroinflammatory responses, oxidative stress, and disturbances in neurotransmitter homeostasis. Studies suggest that these alterations may affect hippocampal structure and function, thereby contributing to impaired learning and memory processes. As a key brain region involved in cognition and stress regulation, the hippocampus may be implicated in the neurobiological mechanisms underlying cognitive dysfunction in ME/CFS. This review integrates current evidence on the potential role of HPA axis dysregulation and related neurobiological alterations in chronic stress-associated cognitive dysfunction in ME/CFS, with the aim of providing a theoretical basis for identifying potential intervention targets and informing strategies centered on HPA axis regulation.

## 1 Introduction

Myalgic encephalomyelitis/chronic fatigue syndrome (ME/CFS) is a disabling chronic multisystem disorder (Fukuda et al., 1994). It is characterized by persistent fatigue not alleviated by rest and accompanied by a spectrum of somatic and central nervous system-related symptoms (Komaroff, 2019). These symptoms substantially impair daily functioning and work capacity, imposing a substantial public health burden (Clayton, 2015). The global prevalence of ME/CFS is estimated to be approximately 1%, affecting 17–24 million individuals worldwide (Lim et al., 2020). However, owing to inconsistencies in case definitions and diagnostic criteria, prevalence estimates vary substantially across studies, and the true disease burden may be underestimated (Brurberg et al., 2014).

Although the clinical manifestations of ME/CFS are highly heterogeneous, cognitive complaints are among its common and disabling features (Aoun Sebaiti et al., 2022). Up to 89% of patients report problems with memory and attention, and these cognitive difficulties are often described subjectively as “brain fog” (Cockshell and Mathias, 2010). Although the exact mechanisms underlying cognitive dysfunction remain unclear, accumulating evidence supports an association between ME/CFS and cognitive deficits (Robinson et al., 2019; Lacasa et al., 2023). Neurocognitive testing in ME/CFS has shown that patients exhibit deficits in information processing speed, attention, concentration, and working memory (Cvejic et al., 2016; Sandoval et al., 2025).

Chronic stress has been proposed as an important contributing factor in the onset and progression of ME/CFS (Salit, 1997; Nater et al., 2011; Lattie et al., 2012). Chronic stress may contribute to HPA axis dysregulation in patients with ME/CFS, thereby altering stress responsivity and potentially contributing to cognitive symptoms (Papadopoulos and Cleare, 2011; Bansal et al., 2025). In addition, the hippocampus, a key brain region involved in learning, memory, and stress regulation, is particularly sensitive to prolonged stress exposure (Kim et al., 2015). It is also a critical region involved in the negative-feedback regulation of the HPA axis, making it especially relevant to the link between chronic stress and cognitive dysfunction in ME/CFS. Experimental studies have shown that chronic stress can disrupt hippocampal structure and plasticity, thereby impairing learning and memory processes (Kim and Diamond, 2002; McLaughlin et al., 2007).

**FIGURE 1:**
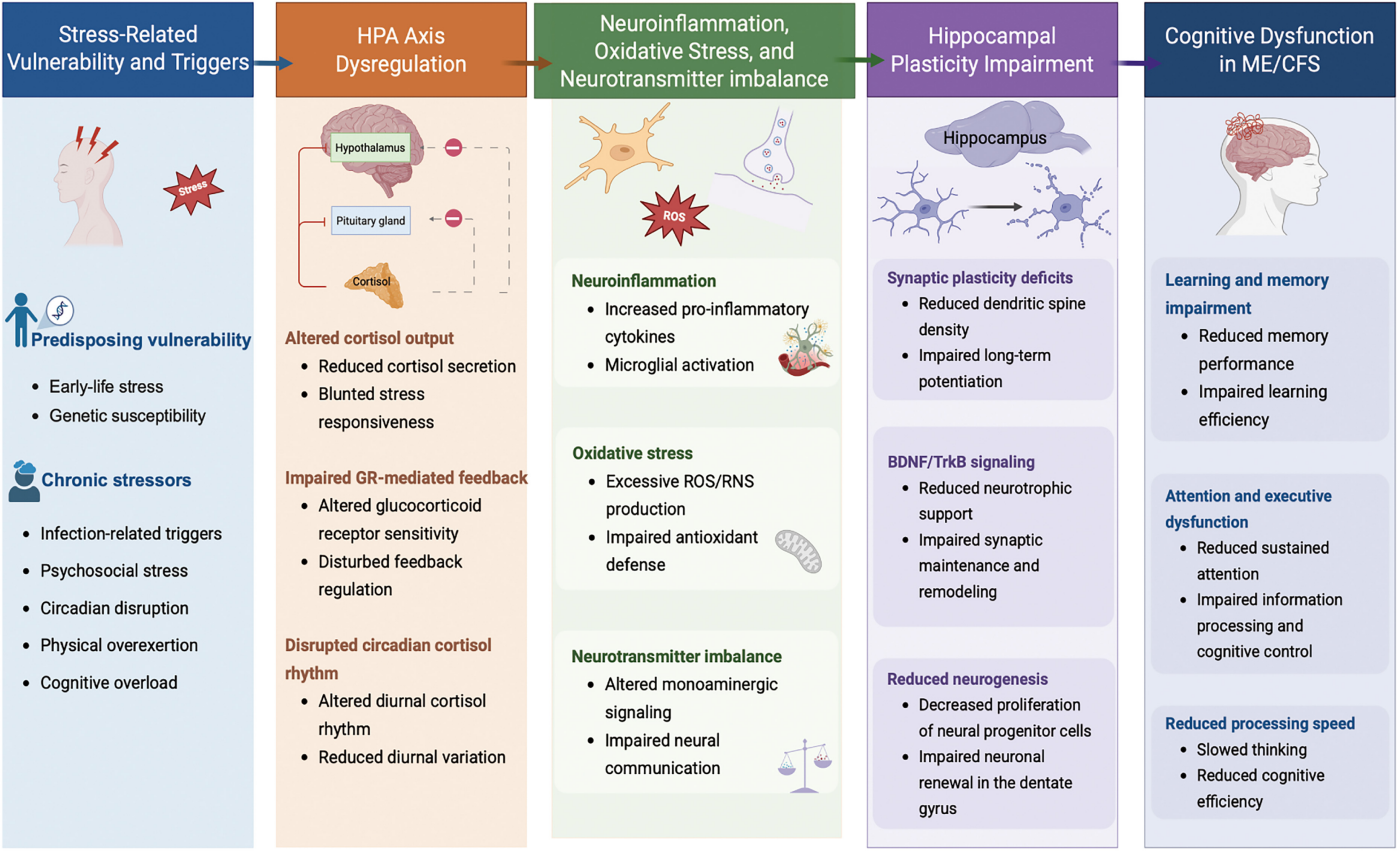
Proposed mechanistic framework linking chronic stress to cognitive dysfunction in ME/CFS. This conceptual model illustrates how predisposing vulnerability factors, including early-life stress and genetic susceptibility, together with chronic stress-related triggers, may contribute to dysregulation of the HPA axis in ME/CFS. Altered cortisol dynamics, impaired feedback regulation, neuroinflammation, oxidative stress, and neurotransmitter imbalance may in turn disrupt hippocampal plasticity, including neurogenesis, synaptic integrity, and functional circuit stability, thereby contributing to deficits in attention, working memory, executive function, and related cognitive domains. HPA, hypothalamic-pituitary-adrenal.

This review focuses on how chronic stress-related HPA axis dysregulation may contribute to cognitive dysfunction in ME/CFS, with particular emphasis on hippocampal involvement. Although HPA axis abnormalities have been reported in ME/CFS, their relationship with hippocampal alterations and cognitive symptoms remains insufficiently understood. By synthesizing current evidence, this review aims to clarify the potential role of HPA axis dysregulation and hippocampal dysfunction in ME/CFS-related cognitive dysfunction, and to provide insight into potential targeted interventions. To provide an integrated overview of these complex interactions, we propose a conceptual framework (Figure 1).

## 2 Stress

Stress is an integral part of modern life. Most individuals experience periods of stress and recognize its potential adverse effects on health. It is increasingly recognized as an important factor in the pathophysiology of ME/CFS (Chu et al., 2019; Nacul et al., 2020; Liu M. Y. et al., 2024). Some researchers have proposed that ME/CFS may be conceptualized as a maladaptive stress-related disorder. In contemporary neuroscience and medical research, stress is generally defined as a series of neuroendocrine responses that are initiated when the body’s homeostasis is threatened to maintain or restore physiological balance (Tsigos et al., 2000). Stress can be classified into acute and chronic types according to the duration of the stressor and the individual’s response pattern. Acute stress induces rapid and usually transient physiological responses, whereas chronic stress reflects prolonged exposure to unresolved stressors and is more strongly associated with sustained multisystem dysregulation (McEwen, 2004; James et al., 2023). Consistent with the classical framework proposed by Hans Selye, prolonged stress may eventually exhaust adaptive capacity, increasing vulnerability to both somatic and neuropsychiatric dysfunction, including cognitive impairment (Tan and Yip, 2018; Rochette et al., 2023).

**FIGURE 2:**
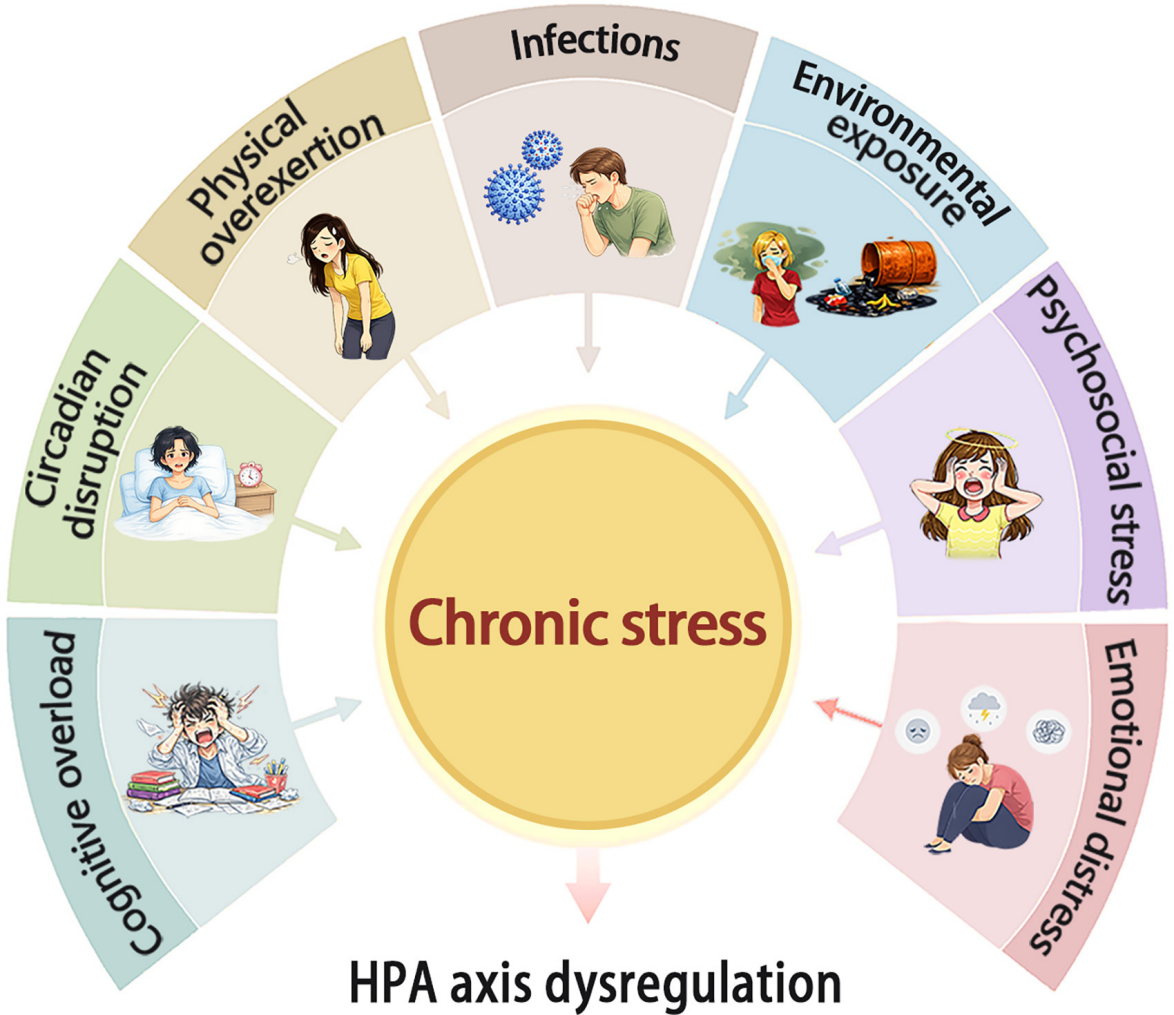
Major stressors contributing to HPA-axis dysregulation in ME/CFS. This schematic illustration summarizes how multiple chronic stress-related factors, including infections, environmental exposure, psychosocial stress, emotional distress, cognitive overload, circadian disruption, and physical overexertion, may converge to promote persistent stress responses and thereby contribute to HPA axis dysregulation in ME/CFS. HPA, hypothalamic-pituitary-adrenal.

The development of ME/CFS is closely associated with stressful life events (Hatcher and House, 2003). Epidemiological surveys and some studies on ME/CFS indicate that many patients experience prolonged mental and physical overexertion, psychological stress, and frequent negative life events prior to disease onset (Theorell et al., 1999; Jason et al., 2000; Jason et al., 2009). These factors are associated with disease development, fatigue severity, and changes in the disease course. In an epidemiological survey conducted in Australia, nearly half of the patients who attributed their illness to noninfectious triggers reported “undue stress” and other life stressors as precipitating factors (Johnston et al., 2016; Balinas et al., 2021; Figure 2). In addition, experiences of victimization during childhood and adulthood play an important role in the onset and progression of ME/CFS (Johnson et al., 2010). Early-life adversity may be particularly relevant because it can produce long-lasting alterations in stress responsivity and physiological homeostasis across the lifespan (Smith and Pollak, 2020). Studies have reported that more than half of patients with ME/CFS reported at least one experience of early childhood trauma (Kempke et al., 2013). It has been estimated that childhood trauma increases the risk of ME/CFS three- to eightfold (Heim et al., 2006; Borsini et al., 2014).

Mechanistically, early adversity and chronic stress may induce persistent biological changes through long-term stress programming and the HPA axis (Heim et al., 2001; Baumeister et al., 2016; Schär et al., 2022). These systems are also among the most consistently implicated in ME/CFS. Over time, unresolved stress may progressively impair the compensatory capacity of neuroendocrine, immune, and autonomic regulatory networks, thereby increasing susceptibility to chronic multisystem dysfunction (Pfaltz and Schnyder, 2023; Park et al., 2025).

## 3 Effect of chronic stress on central fatigue

In ME/CFS, the profound sense of exhaustion is thought to reflect not only peripheral fatigue but also a substantial component of central fatigue, which is difficult to relieve by rest and is often accompanied by reduced motivation and cognitive dysfunction (Neeck and Riedel, 1994; Ocon, 2013; Wan et al., 2017). Neuroimaging findings support the central basis of these symptoms (Maksoud et al., 2020). Several resting-state fMRI studies have reported altered functional connectivity in brain regions involved in cognitive control and emotional processing, including the prefrontal cortex, hippocampal and parahippocampal regions, and the cingulate cortex, in patients with ME/CFS. These abnormalities are significantly correlated with the severity of subjective fatigue (Gay et al., 2016). Task-based MRI studies in ME/CFS have reported a low-efficiency compensatory pattern within cognitive networks, as well as abnormal hemodynamic responses. Overall, these findings reflect decreased cognitive processing efficiency and increased central resource demands (Shan et al., 2020).

Stress and stress hormones exert both adaptive and maladaptive effects on the brain across the lifespan (McEwen, 2007). The brain is both a primary target and a central regulator of the stress response. Higher-order regions, including the prefrontal cortex, hippocampus, and amygdala, are involved in evaluating external stimuli as threats (McEwen and Gianaros, 2011), while the hypothalamus serves as a key hub of neuroendocrine and autonomic regulation, initiating and maintaining the physiological stress response (Herman et al., 2016; Schaeuble and Myers, 2022). Chronic stress is considered a key precipitating factor in central nervous system dysfunction in ME/CFS and may impair brain structure and function through multiple mechanisms. Even before the 1994 Centers for Disease Control and Prevention definition of ME/CFS, Kent-Braun et al. proposed that physical symptoms in ME/CFS are partly related to stressor processing within the central nervous system (Kent-Braun et al., 1993).

The magnitude, duration, source, and intensity of stress can differentially influence cognitive function. Moderate stress may enhance cognitive performance, whereas prolonged and severe stress can lead to cognitive impairment, particularly deficits in memory and judgment (Lin et al., 2022). Human studies have reported comparable findings, showing that specific stressors, such as orthostatic stress and physical or cognitive exertion, can further exacerbate cognitive dysfunction in individuals with ME/CFS (van Campen et al., 2020; Lange et al., 2024). Animal studies likewise support a mechanistic link between chronic stress, central fatigue, and hippocampal dysfunction. A central fatigue model established using the modified multiple platform method combined with alternate-day fasting reproduced several features relevant to ME/CFS, including reduced exploratory behavior, learning and memory impairment, physical fatigue, and hippocampal damage (Zhang et al., 2024).

## 4 Chronic stress affects HPA axis activity and cortisol regulation

**FIGURE 3:**
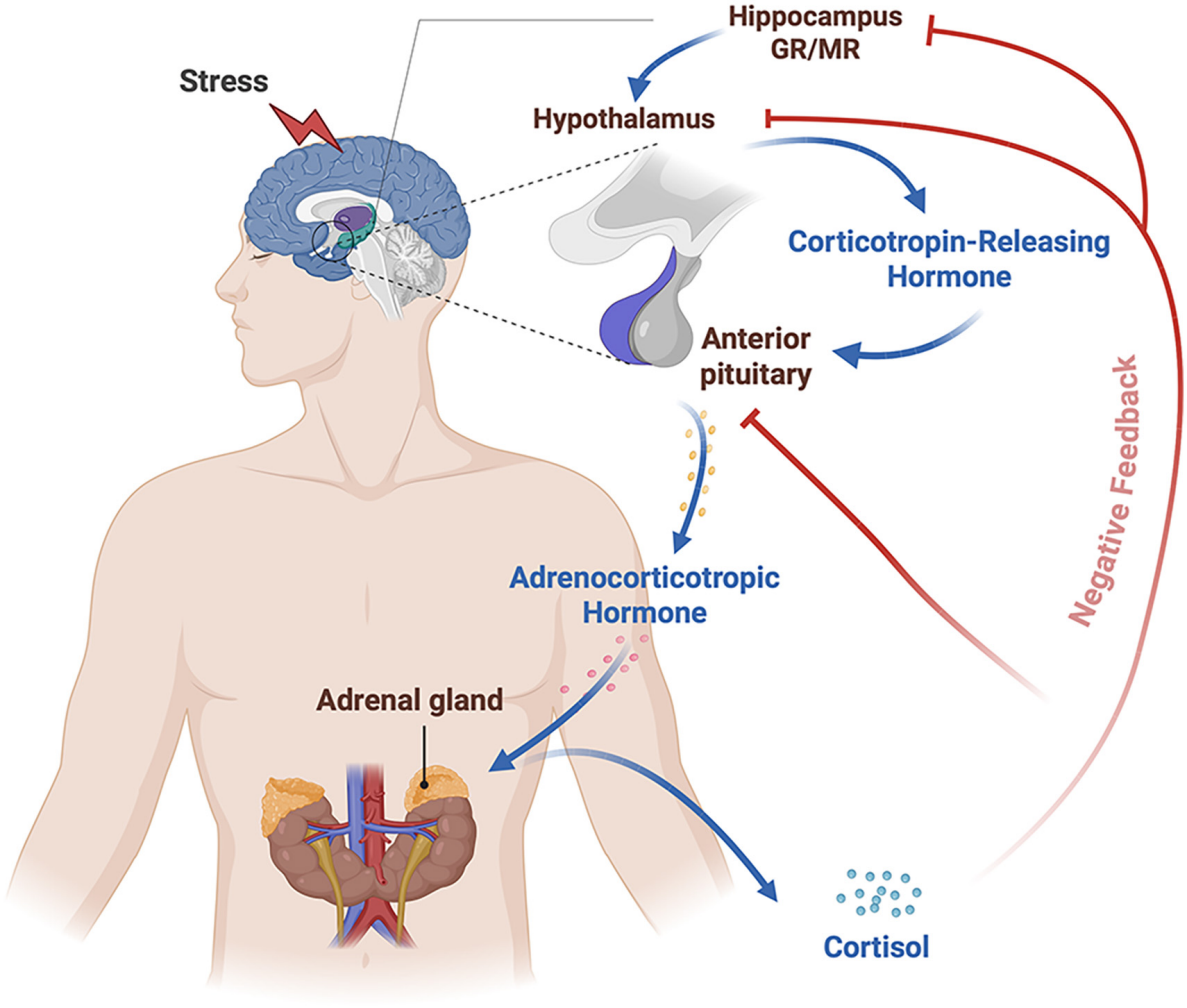
Stress-induced activation of the HPA axis and its negative feedback regulation. This schematic illustrates the canonical neuroendocrine cascade through which perceived stress activates the HPA axis. Stress stimulates the hypothalamus to release CRH, which in turn promotes ACTH secretion from the anterior pituitary and subsequent cortisol release from the adrenal gland. Cortisol then exerts negative feedback at both the hypothalamic and pituitary levels, while the hippocampus contributes indirectly to feedback regulation through GR and MR-related pathways, thereby helping to restrain further HPA-axis activation and maintain neuroendocrine homeostasis. In this schematic, arrows indicate activation, whereas blunt-ended lines indicate inhibitory feedback. HPA, hypothalamic-pituitary-adrenal; CRH, corticotropin-releasing hormone; ACTH, adrenocorticotropic hormone; GR, glucocorticoid receptor; MR, mineralocorticoid receptor.

The HPA axis is a key neuroendocrine system that regulates stress responses and represents one of the core pathways mediating stress reactions (Parker et al., 2001; Smith and Vale, 2006). When the body is exposed to stress, the paraventricular nucleus (PVN) of the hypothalamus secretes corticotropin-releasing hormone (CRH) and arginine vasopressin (AVP), which stimulate the anterior pituitary to release adrenocorticotropic hormone (ACTH) (Heim et al., 2000; Gjerstad et al., 2018). ACTH subsequently acts on the adrenal cortex to induce glucocorticoid secretion (cortisol in humans and corticosterone in rodents) (Dickerson and Kemeny, 2004; Herman et al., 2020). Glucocorticoids are released in a pulsatile manner and exert negative feedback through glucocorticoid receptors (GRs) and mineralocorticoid receptors (MRs) in the hypothalamus, pituitary, and hippocampus, thereby contributing to suppression of further CRH and ACTH secretion and maintenance of HPA-axis homeostasis (Gjerstad et al., 2018; Figure 3).

Basal hypocortisolism was first reported in patients with ME/CFS in 1981 (Poteliakhoff, 1981). Demitrack and colleagues reported (Demitrack et al., 1991) that cortisol excretion in patients with ME/CFS was lower than normal. Later studies have further supported reduced plasma and salivary cortisol levels (Strickland et al., 1998; Roberts et al., 2004; Nater et al., 2008a), a flattened diurnal secretion rhythm (Jerjes et al., 2005; Nater et al., 2008b), and decreased urinary free cortisol excretion (Scott and Dinan, 1998; Cleare et al., 2001; Jerjes et al., 2006) in ME/CFS, indicating that HPA axis hypofunction represents an important neuroendocrine feature of the disorder. Gaab and colleagues systematically assessed (Gaab et al., 2002a) HPA axis reactivity in patients with ME/CFS using three types of stimulation, including Trier Social Stress Test, exercise, and insulin tolerance test. The results showed that ACTH responses to psychological and physiological stress were significantly blunted in patients with ME/CFS, whereas cortisol responses were relatively preserved or only mildly reduced, suggesting reduced central drive within the stress response system. However, cortisol-related findings are not entirely uniform. Although the overall evidence suggests that at least a subset of patients with ME/CFS exhibit reduced cortisol output, some cohorts show only modest reductions, preserved cortisol output, or context-dependent abnormalities rather than stable hypocortisolism, indicating that HPA-axis dysfunction in ME/CFS is likely heterogeneous and multifactorial rather than a specific or uniform neuroendocrine abnormality (Cleare, 2003; Tomas et al., 2013; Taccori et al., 2023). Accordingly, HPA-axis hypofunction may be better viewed as a common but not universal neuroendocrine pattern in ME/CFS.

Since 1998, Scott and colleagues have regarded ME/CFS as a stress-related disorder and proposed that HPA-axis hypofunction may reflect a state of stress-related hypocortisolism or reduced HPA-axis drive (Scott et al., 1998). In the initial phase of stress, activation of the HPA axis leads to an increase in cortisol levels and simultaneously initiates a negative feedback mechanism (O’Connor et al., 2000). With prolonged or repeated stress exposure, however, the central drive of the HPA axis gradually weakens, and negative feedback regulation becomes dysregulated, resulting in a sustained state of low cortisol levels and blunted stress responsiveness. Evidence for altered negative-feedback regulation has also been reported. Low-dose dexamethasone suppression testing (Gaab et al., 2002b) and prednisolone challenge experiments (Jerjes et al., 2007) both showed greater cortisol suppression in patients with ME/CFS than in healthy controls, supporting abnormal glucocorticoid feedback regulation. In addition, epigenetic studies have identified increased methylation in the promoter region of the glucocorticoid receptor (GR) gene, NR3C1, which may be related to altered receptor regulation and has also been associated with childhood trauma (Vangeel et al., 2015; Vangeel et al., 2018). Overall, substantial evidence supports HPA axis hypofunction in ME/CFS, although findings remain heterogeneous and the underlying mechanisms are not yet fully understood. Potential explanations include reduced ACTH output (Demitrack et al., 1991; Scott et al., 1998), reduced adrenal size (Scott et al., 1999a; Scott et al., 1999b), a compensatory shift from HPA axis hyperresponsiveness to hyporesponsiveness following chronic stress (Fries et al., 2005; Cortes Rivera et al., 2019), and enhanced negative feedback (Gaab et al., 2005; Arnett et al., 2011).

In ME/CFS, it remains unclear whether neuroendocrine abnormalities reflect predisposing vulnerabilities, secondary consequences of prolonged disease burden, or context-dependent adaptations. This uncertainty likely reflects both the clinical heterogeneity of the disorder and methodological differences across studies, including variation in case definitions, illness duration, symptom severity, comorbidities, medication exposure, and sampling protocols (Tanriverdi et al., 2007; Lim and Son, 2020; Nacul et al., 2020). In addition, several illness-related factors may themselves influence HPA-axis function, and the greater prominence of HPA-axis abnormalities in patients with longer illness duration supports the possibility that some endocrine changes are secondary (Cleare, 2004). Such changes may also contribute to symptom persistence, suggesting that HPA-axis dysregulation in ME/CFS may be better understood as both a contributing factor and a consequence of disease progression rather than a strictly unidirectional mechanism.

The hippocampus is especially relevant in this context because it is both highly stress-sensitive and critically involved in the negative-feedback regulation of the HPA axis. GC production and secretion are tightly regulated by feedback mechanisms, primarily mediated by cortisol binding to glucocorticoid receptors (GR) and mineralocorticoid receptors (MR), which initiate negative feedback inhibition at multiple levels of the central nervous system and the HPA axis (De Kloet et al., 1998). GR predominantly mediates feedback regulation under conditions of elevated circulating cortisol, whereas MR is mainly involved in basal feedback control (de Kloet et al., 2005). In the brain, both receptors are highly expressed in the hippocampus, highlighting its particular sensitivity to stress (Watzka et al., 2000). As a key limbic region involved in HPA-axis feedback regulation, the hippocampus indirectly modulates hypothalamic CRH neuron activity through GR- and MR-related regulatory pathways, thereby contributing to neuroendocrine homeostasis during long-term adaptation to stress (Herman et al., 2003).

## 5 Alterations in the hippocampus due to chronic stress

A large body of evidence has consistently shown that stress can markedly alter hippocampal structure and function and thereby impair hippocampus-dependent memory (Fuchs and Flügge, 1998; Anand and Dhikav, 2012; Zhang et al., 2020; Shin et al., 2024). However, evidence directly linking chronic stress to changes in hippocampal plasticity in ME/CFS remains limited. The hippocampus not only plays a pivotal role in maintaining HPA axis homeostasis but is also a major target of stress-related damage. Prolonged chronic stress and dysregulated glucocorticoid levels can disrupt the hippocampal neural circuitry, with characteristic structural alterations, including dendritic atrophy and remodeling, reduced dendritic spine density, and suppressed adult neurogenesis.

### 5.1 Chronic stress and hippocampal synaptic plasticity

Synaptic plasticity refers to changes in synaptic structure and function in response to neuronal activity and serves as a fundamental neurobiological basis for learning and memory (Takeuchi et al., 2014). It is primarily manifested as bidirectional changes in synaptic strength, most notably long-term potentiation (LTP) and long-term depression (LTD) (Malenka and Bear, 2004; Bliss and Cooke, 2011). Studies have shown that stress and GCs can drive dendritic atrophy and spine loss in the hippocampus (Chen Y. et al., 2008; Liston and Gan, 2011). Such structural alterations may weaken neural connectivity and flexibility, thereby impairing learning and memory processes.

Early evidence linking stress to hippocampal synaptic plasticity came from electrophysiological studies showing that intermittent tail shock during restraint significantly impaired LTP in the Schaffer collateral-commissural pathway to the cornu ammonis 1 (CA1) region in rat hippocampal slices (Foy et al., 1987). This finding provided early evidence that stress can directly disrupt hippocampal synaptic plasticity and thereby interfere with learning and memory. More recently, Chuwen Feng and colleagues established a rat model of ME/CFS using a 35-day chronic multifactorial stress paradigm. Histopathological examination revealed marked structural alterations and synaptic ultrastructural damage in the hippocampal CA1 region, while proteomic analysis identified differentially expressed proteins enriched in pathways related to synaptic plasticity, neurotransmitter release, and associated signaling cascades (Chuwen et al., 2025). These findings further support the idea that chronic stress-like conditions may induce broad remodeling of hippocampal plasticity-related protein networks.

**FIGURE 4:**
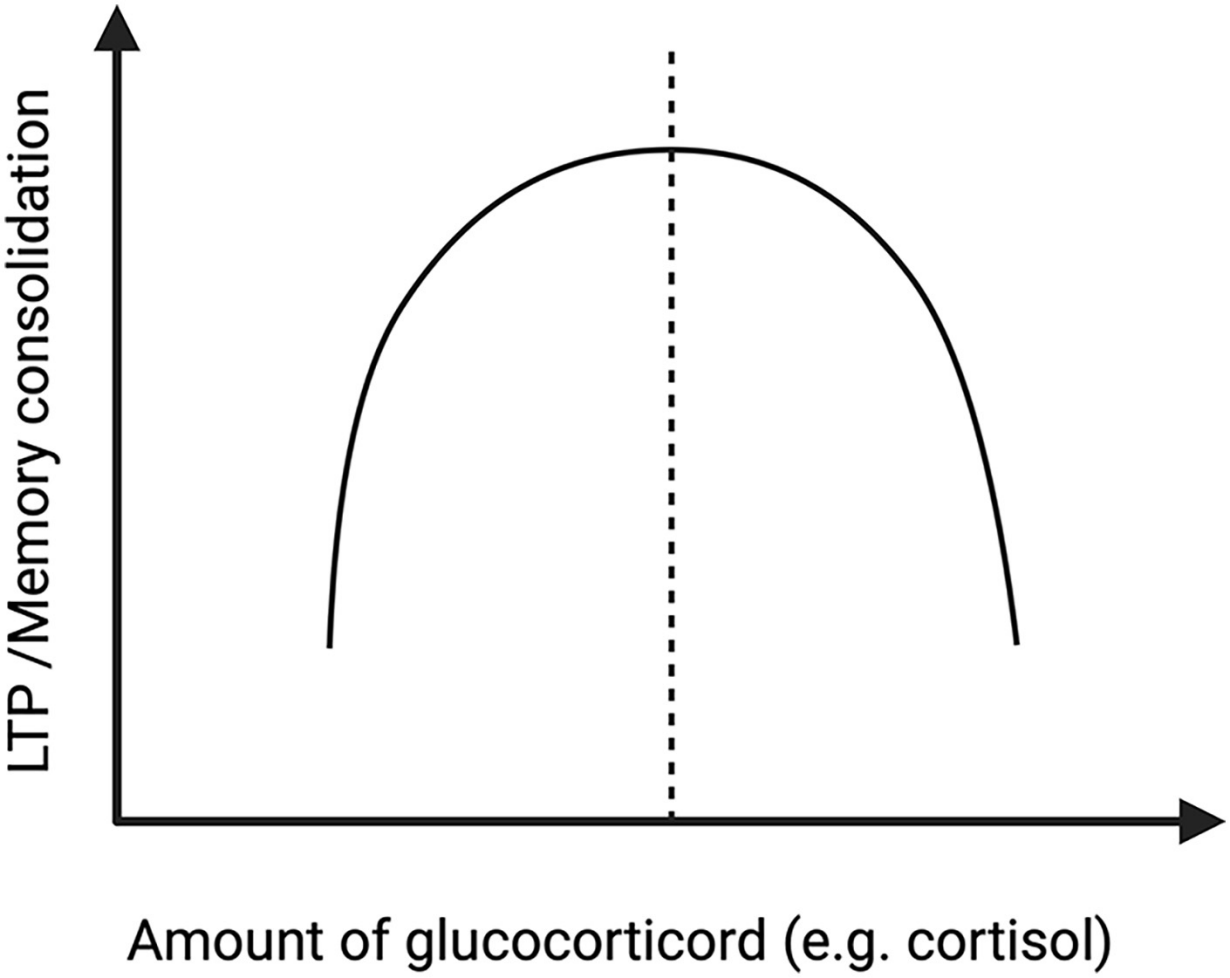
Inverted U-shaped relationship between glucocorticoid levels and hippocampal LTP. This schematic graph illustrates the nonlinear relationship between glucocorticoid signaling and hippocampal synaptic plasticity. The horizontal axis represents the relative level of glucocorticoid exposure, whereas the vertical axis represents the degree of LTP or memory consolidation. The inverted U-shaped model indicates that an intermediate range of glucocorticoid signaling is most favorable for hippocampal plasticity, while both insufficient and excessive glucocorticoid exposure may impair LTP and memory-related processes. LTP, long-term potentiation.

Although chronically elevated cortisol is often considered detrimental, insufficient glucocorticoid signaling may also impair synaptic plasticity. Evidence from animal studies suggests that the effects of GCs on LTP and memory follow an inverted U-shaped dose-response relationship, with moderate physiological levels supporting synaptic plasticity and memory consolidation, whereas both insufficient and excessive GC exposure are associated with impairment (Pavlides et al., 2002; Liu M. Y. et al., 2024; Figure 4).

**FIGURE 5:**
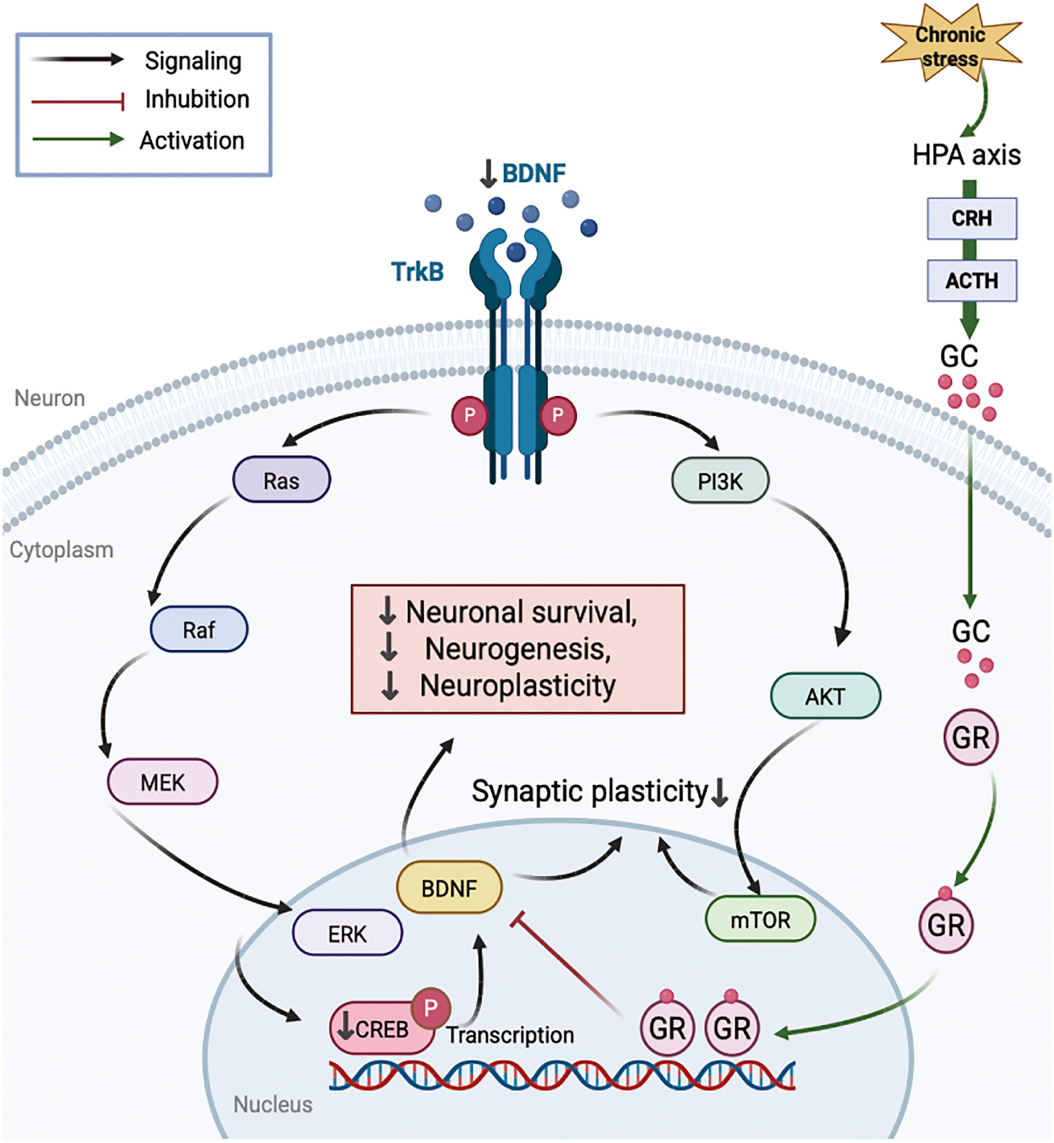
Proposed effects of stress-related glucocorticoid dysregulation on BDNF/TrkB-mediated neuroplasticity. This schematic illustrates a proposed mechanism by which stress-related dysregulation of the HPA axis may alter GC signaling and GR-mediated regulation, potentially leading to impaired BDNF/TrkB-dependent neuroplastic signaling. This may attenuate downstream PI3K/Akt/mTOR and Ras/Raf/MEK/ERK pathways, decrease CREB phosphorylation, and ultimately contribute to reduced synaptic plasticity, neuronal survival, and neurogenesis. In the diagram, black arrows indicate signaling flow, green arrows indicate activation, and red blunt-ended lines indicate inhibition. HPA, hypothalamic-pituitary-adrenal; CRH, corticotropin-releasing hormone; ACTH, adrenocorticotropic hormone; GC, glucocorticoid; GR, glucocorticoid receptor; BDNF, brain-derived neurotrophic factor; TrkB, tropomyosin receptor kinase B; CREB, cAMP response element-binding protein; PI3K, phosphoinositide 3-kinase; Akt, protein kinase B; mTOR, mechanistic target of rapamycin; ERK, extracellular signal-regulated kinase.

The brain-derived neurotrophic factor (BDNF) pathway is considered an important molecular link between GC signaling and LTP regulation. Both excessive and insufficient GC signaling may disturb BDNF expression and related neuroplastic processes in the hippocampus (Suri and Vaidya, 2013). BALB/c mice injected six times with inactivated Brucella abortus antigen exhibited a significant decrease in central nervous system BDNF mRNA expression, suggesting reduced neuroprotective capacity and impaired synaptic plasticity (Chen R. et al., 2008). BDNF is a key regulator of neural plasticity, promoting synaptic strengthening, neuronal survival, and synaptogenesis, with particularly important effects in hippocampal regions involved in learning and memory (Numakawa et al., 2013). Under conditions of chronically low corticosterone, insufficient MR/GR activation may contribute to reduced BDNF transcription through impaired cAMP response element-binding protein (CREB)-dependent regulation. Reduced BDNF availability may in turn limit TrkB phosphorylation and attenuate downstream PI3K/Akt and MAPK/ERK signaling (Numakawa and Kajihara, 2023). Disruption of these signaling cascades may lead to reduced expression of synapse-associated proteins (Yoshii and Constantine-Paton, 2007). Collectively, these alterations may weaken excitatory synaptic transmission and compromise the induction and maintenance of hippocampal LTP (Figure 5). However, much of this mechanistic framework is derived from experimental and animal studies, and direct validation in patients with ME/CFS remains limited.

### 5.2 Chronic stress and adult hippocampal neurogenesis

**FIGURE 6:**
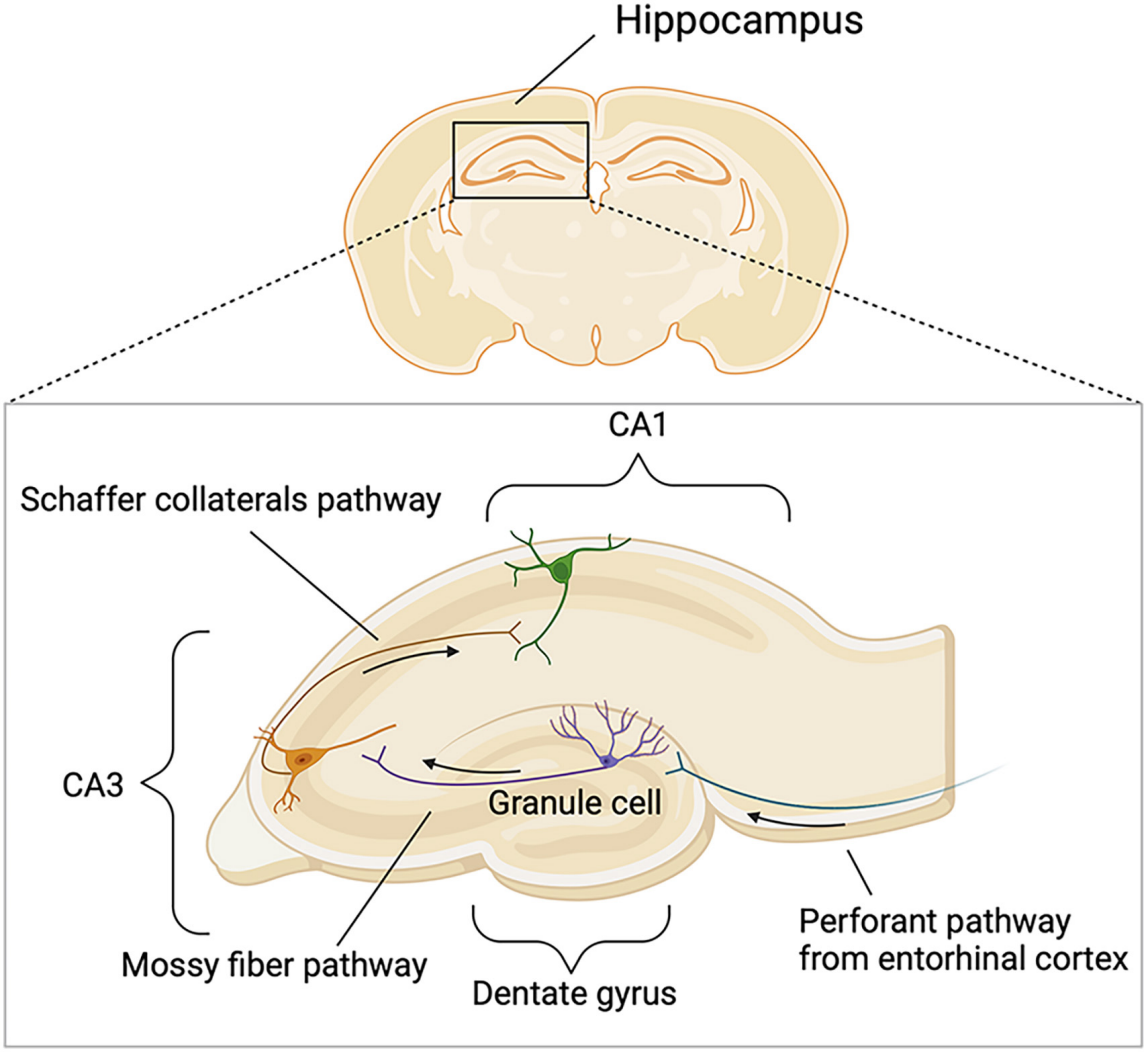
Schematic representation of the hippocampal trisynaptic circuit. This schematic illustrates the major anatomical organization of the hippocampal trisynaptic circuit. The upper panel indicates the location of the hippocampus in a coronal brain section, and the lower panel depicts the principal intrahippocampal pathways. Afferent input from the entorhinal cortex reaches DG granule cells through the perforant pathway. DG mossy fibers then project to CA3 pyramidal neurons, which in turn send Schaffer collateral projections to CA1 pyramidal neurons. Arrows indicate the predominant direction of signal transmission within the trisynaptic circuit. DG, dentate gyrus; CA3, cornu ammonis 3; CA1, cornu ammonis 1.

The hippocampus serves as a critical hub for stress regulation and cognitive processing and is therefore highly relevant to cognitive dysfunction in disorders such as ME/CFS. Its highly organized laminar circuitry and subregional specialization confer particular vulnerability to chronic stress and glucocorticoid dysregulation. Information from neocortical association areas enters the hippocampus via the entorhinal cortex (EC) and propagates along the classical trisynaptic circuit (Figure 6). Within this circuit, the dentate gyrus (DG) supports pattern separation, cornu ammonis 3 (CA3) contributes to pattern completion, and CA1 integrates hippocampal input for downstream output processing. In addition to its role in memory processing, hippocampal output through the subiculum is anatomically linked to hypothalamic pathways, providing a structural basis for interactions between hippocampal function and stress-axis regulation (Basu and Siegelbaum, 2015).

Beyond its well-characterized circuit organization, the hippocampus retains a capacity for neurogenesis. Hippocampal neurogenesis mainly occurs in the subgranular zone (SGZ) of the dentate gyrus, where newborn neurons are continuously generated and integrated into existing circuits, thereby contributing to hippocampal plasticity, learning, and memory. Importantly, direct histological evidence demonstrating impaired hippocampal neurogenesis in patients with ME/CFS is currently lacking. Most existing evidence is derived from animal models or inferred from related disorders, which limits direct extrapolation to human ME/CFS. Therefore, whether similar alterations occur in patients with ME/CFS remains to be established.

GCs exert dose-dependent biphasic effects on hippocampal plasticity. Adrenalectomy and graded corticosterone replacement studies have shown that prolonged exposure to extremely low GC levels can induce dendritic atrophy and reduced structural complexity in CA3 pyramidal neurons (Martínez-Claros et al., 2013). In contrast, replacement with moderate physiological doses of GCs appears to prevent degenerative changes and support the survival and integration of newborn neurons in the DG. These findings indicate that GC regulation of hippocampal plasticity does not follow a simple linear relationship but instead operates within an optimal hormonal window that supports dendritic stability and sustained neurogenesis.

Given the close interactions among stress, GC signaling, and hippocampal neurogenesis, ME/CFS-related conditions characterized by chronic stress exposure and HPA-axis dysregulation may plausibly involve vulnerability in neurogenic processes and related plasticity networks. Animal models provide partial support for this possibility. In a model induced by repeated Brucella antigen injections, hippocampal atrophy was accompanied by reduced DG neurogenesis, increased granule-cell apoptosis, and decreased BDNF expression (Moriya et al., 2011). Resveratrol ameliorated these alterations by enhancing neurogenesis and suppressing apoptosis. However, another study using a comparable model reported elevated cytokine and oxidative stress markers in the cortex and hippocampus without detectable changes in neurogenesis (He et al., 2020). These findings suggest that neurogenic alterations in ME/CFS-related models may vary with model design and timing, and that suppressed neurogenesis should not yet be regarded as a uniform feature across all fatigue paradigms.

## 6 Mechanisms of chronic stress-induced neuronal dysfunction

### 6.1 Neuroinflammation

Neuroinflammation refers to immune activation and inflammatory responses within the central nervous system (CNS), typically involving sustained activation of microglia and astrocytes as well as excessive release of proinflammatory cytokines (DiSabato et al., 2016; Kölliker-Frers et al., 2021). Neuroinflammation may represent an important pathological component in the onset and progression of ME/CFS (Hornig et al., 2015). Early studies reported stage-related immunological abnormalities in ME/CFS, including impaired natural killer cell function (Sotzny et al., 2018), increased numbers of activated CD8^+^ cytotoxic T cells (Rivas et al., 2018), and elevated levels of multiple proinflammatory cytokines (Montoya et al., 2017). Clinical and biofluid studies have further reported altered cytokine profiles in both peripheral blood and cerebrospinal fluid, suggesting the coexistence of systemic and central inflammatory processes in ME/CFS (Hornig et al., 2016; Montoya et al., 2017; Bastos et al., 2025). Sustained inflammatory signaling in peripheral compartments or CSF may also be associated with, and potentially exacerbate, cognitive dysfunction (Montoya et al., 2017; Mekhora et al., 2024).

Neuroimaging studies provide additional support for central neuroinflammation in ME/CFS. Using whole-brain MRS/EPSI, Mueller and colleagues quantified metabolite ratios across 47 brain regions and reported associations between several glial and metabolism-related metabolites and fatigue severity (Mueller et al., 2020). These associations involve metabolites often linked to glial activity and membrane and energy metabolism, including choline (Cho), myo-inositol (mIns), lactate (LAC), and N-acetylaspartate (NAA). More direct *in vivo* evidence has come from positron emission tomography (PET) studies using the translocator protein (TSPO) tracer ^11^C-(R)-PK11195. Compared with healthy controls, patients with ME/CFS showed increased TSPO binding in multiple brain regions, including the hippocampus, amygdala, and thalamus, findings consistent with elevated glial activity. TSPO binding in selected regions was also associated with worse cognitive performance, suggesting a potential link between central glial activation and cognitive dysfunction (Nakatomi et al., 2014).

The nervous, endocrine, and immune systems interact bidirectionally to maintain physiological homeostasis during inflammatory responses and cytokine production. The relationship between chronic stress and cytokine responses is dynamic and multifaceted (Alotiby, 2024). Current evidence suggests that chronic stress is often accompanied by elevated levels of proinflammatory cytokines, including IL-6, TNF-α, and IL-1β. These inflammatory signals can reach the brain through humoral pathways and neural afferent routes, potentially influencing HPA-axis activation and regulation (Dantzer et al., 2000). During the early phase of chronic stress, the HPA axis may be transiently activated, leading to elevated circulating cortisol levels that suppress proinflammatory cytokine production through glucocorticoid receptor-mediated negative feedback (Alotiby, 2024). However, with prolonged or repeated stress exposure, glucocorticoid resistance and altered feedback regulation may emerge, resulting in reduced HPA-axis responsiveness and attenuation of cortisol’s immunosuppressive effects. This may permit sustained elevations in proinflammatory cytokines and disruption of immune homeostasis (Morris et al., 2017a). In addition, TNF-α, IL-1β, and IL-6 can stimulate each other’s production and act synergistically through positive feedback loops, thereby amplifying inflammatory signaling (Donzis and Tronson, 2014; Bourgognon and Cavanagh, 2020).

**FIGURE 7:**
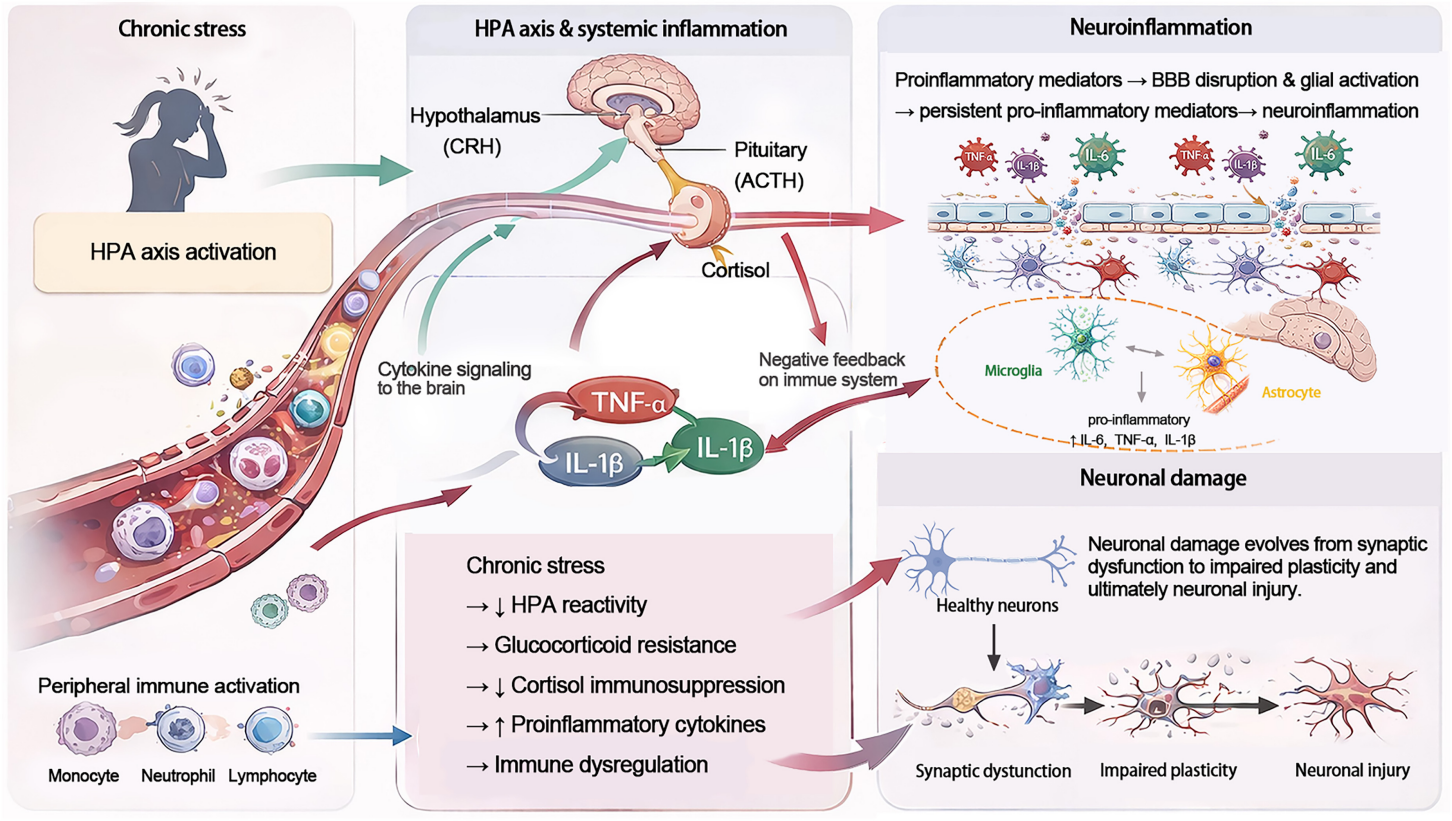
Chronic stress-related HPA axis dysregulation, systemic inflammation, and neuroinflammatory injury in ME/CFS. This schematic illustrates a proposed mechanism by which chronic stress contributes to HPA axis dysregulation and peripheral immune imbalance in ME/CFS. In the left and central panels, chronic stress is associated with altered HPA axis activity, reduced cortisol-mediated immunosuppression, glucocorticoid resistance, and increased production of pro-inflammatory cytokines, including TNF-α, IL-1β, and IL-6. These circulating inflammatory mediators may signal to the brain and promote BBB disruption and glial activation. In the upper right panel, activated microglia and astrocytes further amplify neuroinflammation through sustained release of pro-inflammatory mediators. In the lower right panel, persistent neuroinflammation contributes to synaptic dysfunction, impaired neuroplasticity, and progressive neuronal injury. HPA, hypothalamic-pituitary-adrenal; BBB, blood-brain barrier; CRH, corticotropin-releasing hormone; ACTH, adrenocorticotropic hormone; TNF-α, tumor necrosis factor-α; IL-1β, interleukin-1β; IL-6, interleukin-6.

Cytokines are also important regulators of blood-brain barrier (BBB) integrity. Elevated circulating proinflammatory cytokines and activated peripheral immune cells may directly or indirectly impair BBB function and increase its permeability (Galea, 2021). Subsequently, infiltrating inflammatory mediators and immune cells can trigger reactive activation of microglia and astrocytes, promoting further inflammatory signaling within the CNS and thereby sustaining a neuroinflammatory microenvironment (Linnerbauer et al., 2020; Figure 7). Early histological studies suggested that microglia are relatively abundant in the hippocampus and may mount particularly rapid and robust proinflammatory responses following peripheral immune challenge (Lawson et al., 1990). Microglia are not merely immune surveillance cells; they directly interact with synapses and neurons and secrete cytokines and neurotrophic factors. Through these mechanisms, they play important roles in regulating synaptic plasticity, neurogenesis, and learning and memory processes (Cornell et al., 2022). Accordingly, sustained microglial activation may represent an important link between neuroinflammatory signaling and hippocampus-dependent cognitive dysfunction in ME/CFS.

### 6.2 Oxidative stress

Redox imbalance has been recognized as one of the key pathological features of ME/CFS (Richards et al., 2000; Kennedy et al., 2005). It is characterized by an imbalance between excessive production of reactive oxygen species (ROS) and weakened antioxidant defenses, leading to persistent oxidative stress (Maes, 2009; Lee et al., 2018; Wood et al., 2021). Lipid peroxidation (LPO), a major form of oxidative damage, may contribute to the onset and progression of ME/CFS (Maes et al., 2009b; Brkic et al., 2010; Shankar et al., 2025). Prolonged stress exposure may promote oxidative damage, in part through sustained dysregulation of the HPA axis. Because cortisol is an important regulator of redox homeostasis, reduced cortisol availability or altered glucocorticoid signaling may weaken anti-inflammatory and antioxidant defenses, thereby contributing to persistent oxidative stress.

Previous studies have suggested that oxidative and nitrosative stress are persistently elevated in patients with ME/CFS and may also involve the central nervous system (Morris and Maes, 2014; Arron et al., 2024). As a high-energy organ, the CNS may be particularly susceptible to oxidative stress, and the hippocampus appears to be especially vulnerable to oxidative insults and impaired antioxidant defenses (Wang and Michaelis, 2010). Excessive ROS can disrupt synaptic function, perturb calcium homeostasis, and damage mitochondrial DNA (Massaad and Klann, 2011). While physiological levels of ROS contribute to the induction and maintenance of hippocampal LTP, excessive accumulation may impair neurotransmission and cognitive processing. Given the relatively limited endogenous antioxidant capacity of the brain, elevated ROS levels are more likely to elicit neurotoxic effects. To maintain redox homeostasis, neurons rely on antioxidant systems such as superoxide dismutase (SOD), catalase (CAT), and glutathione peroxidase (GPx) to neutralize free radicals. These defense systems have also been reported to be functionally impaired in ME/CFS (Oktyabrsky and Smirnova, 2007). Alterations in several antioxidant and oxidative stress-related markers reported in ME/CFS are summarized in Table 1.

**TABLE 1:** Oxidative stress-related markers reported in ME/CFS.

Marker	ME/CFS change	Category	Physiological function
Superoxide dismutase (SOD) (Fan et al., 2025; Shankar et al., 2025)	Decreased expression and activity	Antioxidant enzyme	Catalyzes the dismutation of superoxide anions into hydrogen peroxide and oxygen, forming the first enzymatic defense against oxidative stress and protecting mitochondrial function (Maes et al., 2011; Wood et al., 2021).
Malondialdehyde (MDA) (Manuel y Keenoy et al., 2001; Richards et al., 2007; Tomic et al., 2012)	Increased	Lipid peroxidation marker	End product of polyunsaturated fatty acid lipid peroxidation; serves as a marker of membrane lipid oxidative damage (Cui et al., 2018; Davis et al., 2025).
Glutathione (GSH) (Shungu et al., 2012)	Decreased cortical GSH levels	Redox buffer	Major intracellular nonenzymatic antioxidant and redox buffer that maintains cellular redox homeostasis and detoxifies peroxides (Kurup and Kurup, 2003; Kennedy et al., 2005).
Homocysteine (Hcy) (Regland et al., 1997)	Elevated in cerebrospinal fluid	Pro-oxidant metabolic factor	Sulfur-containing intermediate of one-carbon metabolism involved in methylation reactions and redox-related metabolic processes (Kamath et al., 2006; Beard et al., 2011; Kamat et al., 2015).
Nitric oxide (NO) (Kurup and Kurup, 2003; Maes et al., 2006; Morris et al., 2017b)	Increased	Nitrosative stress mediator	Gaseous redox signaling molecule involved in vascular regulation, immune modulation, and cellular signaling; can form reactive nitrogen species under oxidative conditions (Morris and Maes, 2014).
Coenzyme Q (CoQ10) (Maes et al., 2009a; Castro-Marrero et al., 2015; Fukuda et al., 2016; Castro-Marrero et al., 2021; Zhang et al., 2024)	Reduced levels	Lipid-soluble antioxidant	Lipid-soluble component of the mitochondrial respiratory chain that facilitates electron transport, supports ATP synthesis, and functions as an antioxidant (Maes et al., 2009a).
Thiobarbituric acid reactive substances (TBARS) (Vecchiet et al., 2003; Fenouillet et al., 2016; Jammes and Retornaz, 2019)	Increased	Lipid peroxidation marker	Represents thiobarbituric acid-reactive products generated during lipid peroxidation; commonly used as an index of oxidative damage (Natelson, 2013; Bansal et al., 2025).
8-iso-prostaglandin F2α (8-iso-PGF2α) (Kennedy et al., 2005)	Increased	Lipid peroxidation marker	Stable end product of ROS-mediated lipid peroxidation derived from arachidonic acid; widely used as a biomarker of oxidative stress (Liu et al., 2009).
NAD (NAD+/NADH) (Castro-Marrero et al., 2021; Heng et al., 2025)	Imbalance in NAD+/NADH ratio	Redox state regulator	Central cellular redox couple that regulates oxidative metabolism, mitochondrial electron transport, and energy production (Sweetman et al., 2020).

Sustained systemic oxidative stress may in turn contribute to mitochondrial dysfunction, activation of inflammatory pathways, and dysregulation of neural function, thereby potentially contributing to the symptom burden of ME/CFS (Holden et al., 2020; Fineberg et al., 2025). Available evidence supports a bidirectional interplay among elevated oxidative stress, mitochondrial dysfunction, and neuroinflammation (Peggion et al., 2024; Qin et al., 2024). As a central hub for cellular energy metabolism, mitochondria not only maintain cellular homeostasis by regulating ATP production and apoptosis but also modulate immune and inflammatory responses via ATP-mediated purinergic signaling (Picca et al., 2020; Tate et al., 2022; Syed et al., 2025). Under neuroinflammatory conditions, persistently activated microglia can release high levels of ROS and reactive nitrogen species (RNS), further compromising mitochondrial integrity and function (Paul et al., 2021; Tate et al., 2022; Peruzzotti-Jametti et al., 2024). Collectively, this pathological crosstalk may contribute to hippocampal dysfunction and cognitive impairment in ME/CFS.

### 6.3 Neurotransmitter systems

Abnormalities in neurotransmitter systems, particularly imbalances in serotonergic (5-HT) and noradrenergic (NE) pathways, have been linked to chronic stress and may contribute to cognitive impairment in ME/CFS. As early as the late 1980s, Newsholme and colleagues proposed that the central neurotransmitter 5-HT may serve as a key mediator of central fatigue (Newsholme and Blomstrand, 1995). Based on subsequent clinical observations, a hyperserotonergic hypothesis was proposed, suggesting that increased central serotonergic activity may contribute to the pathophysiology of ME/CFS (Meeusen et al., 2006; Lee et al., 2024). More recent studies have reported alterations in monoamine metabolism in the cerebrospinal fluid and plasma of individuals with ME/CFS, particularly elevated levels of the serotonin metabolite 5-hydroxyindoleacetic acid (5-HIAA), suggesting increased central serotonergic turnover (Demitrack et al., 1992; Ferrero et al., 2017). In addition, a PET study showed that patients with ME/CFS exhibited significantly reduced whole-brain 5-HT1A receptor binding potential compared with healthy controls, including an approximately 23% reduction in the bilateral hippocampus, suggesting decreased receptor density or affinity. These findings support the possibility that serotonergic alterations are involved in the pathophysiology of ME/CFS (Yamamoto et al., 2004; Cleare et al., 2005).

5-HT may participate in the stress response by modulating central neuroendocrine networks, particularly through its influence on HPA-axis activity. Under external stress conditions, serotonergic neurons in the dorsal raphe nucleus (DRN) can be activated and release 5-HT; through widespread ascending projections, this signal contributes to the integration and regulation of central stress responses (Liposits et al., 1987; Heisler et al., 2007). Serotonergic inputs to the paraventricular nucleus (PVN) may directly promote CRH release and may also indirectly influence PVN regulatory inputs by modulating neural activity in upstream regions such as the hippocampus, thereby participating in the regulation of HPA-axis function. The 5-HT1A receptor is a key regulatory component of the serotonergic system, functioning both as an autoreceptor that mediates negative feedback on serotonin release and as a heteroreceptor that regulates neuronal activity and neuroendocrine responses (Garcia-Garcia et al., 2014). Prolonged high-intensity stress may lead to sustained serotonergic drive, which can induce desensitization or downregulation of 5-HT1A receptors and thereby impair serotonergic modulation of HPA-axis feedback (Haleem, 2022).

In parallel with serotonergic alterations, abnormalities in the noradrenergic system have also been implicated in ME/CFS (Hendrix et al., 2025). The locus coeruleus (LC), one of the principal sources of central NE, provides dense noradrenergic innervation to the dorsal hippocampus via widespread projections. The LC-NE system is considered a key neuromodulatory pathway linking stress responses with cognitive function and plays an important regulatory role in the encoding, consolidation, and retrieval of hippocampus-dependent memory (Sara, 2009). Under stress conditions, LC activity increases and promotes NE release within the hippocampus, whereas prolonged or repeated stress exposure may disrupt LC-NE signaling and result in blunted noradrenergic responsivity (Slavova et al., 2024). Studies on long-term memory integration further suggest that hippocampal noradrenergic activity modulates the effects of GCs and other neuromodulatory systems on memory processes (Lee et al., 1993). In addition, NE is a critical regulator of hippocampal synaptic plasticity, and its release can modulate long-lasting changes in synaptic efficacy on the basis of LTP and LTD, thereby contributing to memory consolidation and storage (Katsuki et al., 1997; Maity et al., 2015; Palacios-Filardo and Mellor, 2019).

In addition, findings related to neurotransmitter alterations in ME/CFS remain heterogeneous. Some studies suggest serotonergic and noradrenergic abnormalities associated with fatigue and cognitive complaints, whereas others indicate region-specific dysregulation or compensatory changes rather than a uniform directional abnormality (Dinan et al., 1997; Cleare et al., 2005). These inconsistencies may reflect differences in patient characteristics, disease severity, brain-region specificity, and methodological approaches. Importantly, neurotransmitter abnormalities are unlikely to act as isolated defects. Instead, they may interact with HPA-axis dysregulation, neuroinflammatory processes, and hippocampal dysfunction to shape the cognitive symptoms of ME/CFS (Tate et al., 2022; Bansal et al., 2025).

## 7 Therapeutic implications and future directions

At present, there is no curative or approved disease-specific treatment for ME/CFS, and clinical management remains largely supportive, with the primary goals of symptom relief and functional maintenance. According to the latest CDC clinical guidance, current management focuses on post-exertional malaise, sleep disturbance, pain, orthostatic intolerance, and cognitive difficulties, together with supportive care for coexisting anxiety, depression, and stress-related symptoms (Unger et al., 2016). Activity management (pacing) is currently regarded as a central component of care, emphasizing the regulation of physical, cognitive, and emotional activities within an individual’s energy envelope in order to reduce post-exertional symptom exacerbation (Sanal-Hayes et al., 2023).

Overall, therapeutic management in ME/CFS still relies heavily on clinical experience and individualized responses, and robust evidence for specific interventions remains limited. Given the neuroendocrine abnormalities reported in ME/CFS, particularly alterations in HPA axis function, glucocorticoid-based interventions were once considered a potential therapeutic approach. However, available studies suggest that although low-dose hydrocortisone may produce short-term symptomatic improvement in some patients, its effects are inconsistent and often unsustained (Peterson et al., 1998; Cleare et al., 1999), while combined hydrocortisone and fludrocortisone therapy has not shown consistent benefit (Blockmans et al., 2003). In addition, glucocorticoid treatment may be associated with adverse effects, including adrenal suppression, and therefore cannot currently be recommended as a routine treatment for ME/CFS. Nevertheless, the HPA axis may still represent a potential target for future therapeutic development. Some studies suggest that some traditional medicine-derived interventions may ameliorate fatigue-related central abnormalities by modulating HPA axis activity, oxidative stress, mitochondrial function, BDNF-related signaling, and monoaminergic neurotransmission (Surapaneni et al., 2012; Kang et al., 2021). However, these findings are derived predominantly from animal models and should be interpreted cautiously in relation to human ME/CFS.

Lifestyle medicine and related non-pharmacological interventions have also attracted attention, including nutritional optimization (Carrasco-Querol et al., 2023), sleep improvement (Mohamed et al., 2023), appropriate physical activity (Zalewski et al., 2019), and stress management (Lopez et al., 2011). Mindfulness-Based Stress Reduction (MBSR), Mindfulness-Based Cognitive Therapy (MBCT), and stress management skills training have shown potential in some studies to alleviate fatigue, improve emotional well-being, and modulate diurnal cortisol rhythms (Stubhaug et al., 2018; Park et al., 2024). However, the overall evidence base remains constrained by small sample sizes, heterogeneous diagnostic criteria, and a relatively high risk of bias. Taken together, current interventions for ME/CFS remain largely supportive rather than disease-modifying, underscoring the need for future studies to clarify whether targeting chronic stress-related neuroendocrine dysregulation and hippocampal dysfunction may offer more effective therapeutic avenues for cognitive impairment in ME/CFS.

## 8 Conclusion

Chronic stress may contribute to cognitive dysfunction in ME/CFS by disrupting HPA-axis homeostasis and adversely affecting key cognitive regions such as the hippocampus. Prolonged or repeated stress exposure has often been associated with altered cortisol dynamics and may precipitate a cascade of neurobiological changes, including increased neuroinflammatory activity, persistent oxidative stress, dysregulation of key neurotransmitter systems, and structural as well as functional abnormalities within the hippocampus. Collectively, these processes provide a plausible mechanistic basis for deficits in attention, working memory, and related cognitive domains observed in individuals with ME/CFS. A clearer delineation of these interacting pathways will be important for advancing our understanding of ME/CFS pathophysiology and for informing more mechanism-based therapeutic strategies targeting chronic stress-related cognitive impairment.
